# Exploring health workers’ experiences of mental health challenges during care of patients with COVID-19 in Uganda: a qualitative study

**DOI:** 10.1186/s13104-021-05707-4

**Published:** 2021-07-26

**Authors:** Choolwe Muzyamba, Ogylive Makova, Geofrey Samukulu Mushibi

**Affiliations:** 1 University of Amsterdam (UvA)/Utrecht University, Bijlmerdreef 702, 1103DS Amsterdam, The Netherlands; 2 AfriSight, Rotterdam, The Netherlands

**Keywords:** Healthcare workers, Mental health, COVID-19

## Abstract

**Objectives:**

The aim of this study is to qualitatively investigate the lived experiences of mental health among frontline health workers providing COVID-19-realted care in Uganda. This study provides insights into the contextual realities of the mental health of health workers facing greater challenges given the lack of adequate resources, facilities and health workers to meet the demand brought about by COVID-19.

**Results:**

All in all, our findings suggest that healthcare workers are under enormous stress during this pandemic, however, in order to effectively respond to the COVID-19 pandemic in Uganda, it is important to understand their challenges and sources of these challenges. The government thus has the reasonability to address most of the sources that were highlighted (long working hours, lack of proper equipment, lack of sleep, exhaustion, and experiencing high death rate under their care). Further, the Ugandan social fabric presents an opportunity for coping through its strong communal links and networks. Scaling these forms of local responses is cheap but contextually useful for a country with limited resources like Uganda.

**Supplementary Information:**

The online version contains supplementary material available at 10.1186/s13104-021-05707-4.

## Introduction

On 11th March 2020, the World Health Organization (WHO) declared COVID-19 a global pandemic. Since then, the rate of infections around the world has increased and, in the process, placing additional stress on health systems and health workers. This has been precipitated in part by the lack of adequate resources, facilities and health workers to meet the demand brought about by COVID-19. Healthcare workers are at the frontline of the pandemic and as such face higher risks of infection. Data from WHO indicate that infection rates among health workers are higher than that of the general population in almost all WHO regions [1]. Health workers not only face the risk of infection, but the pandemic has also led to high levels of psychological stress as they are exposed to long working hours, living in constant fear of disease exposure while being separated from family and facing social stigmatization [1] as well as physical violence in some instances.

Studies coming from the global north countries, including China have shed light on the vulnerability that characterizes frontline health workers during this pandemic especially regarding their mental health and wellbeing [2, 3]. The World Health Organization defines mental health as “a state of well-being in which the individual realizes his or her own abilities, can cope with the normal stresses of life, can work productively and fruitfully, and is able to make a contribution to his or her community”. Mental health challenges have been a defining characteristic of several frontline health workers during this pandemic. Various elements have been identified as risk factors contributing to higher prevalence of mental health challenges, these include: existing comorbidities identified such as asthma and diabetes, lack of protective equipment in health facilities, lacking adequate knowledge of prevention and care, working overtime, and concern about exposing family members to COVID-19 [3]. While evidence of the sources and experience with mental health has been building in the global north, similar evidence is still missing in the global south countries such as Uganda that are facing a bigger strain on their healthcare system. This is due to already overburden healthcare system resulting from various communicable diseases stacked against inadequate and inefficient response. Health care provision in Uganda is limited due to the number of very few health care workers in the country compared to the demand. This is even worse in rural health care centers where hospitals operate with staffing capacity that is less than 50% the required number. Overall, in Uganda health worker density (number of health workers per 1, 000 people) is only about 0.71 representing a huge shortage in care [4]. Such a predicament coupled high infectious diseases like HIV/AIDS, Malaria and lower respiratory infections has put the health care system in Uganda at serious risk of collapse [5]. Therefore, the onset of COVID-19 poses an even greater threat on the health care system particularly on the very few frontline health workers providing care to COVID-19 patients. Thus, evidence of the experience of health care workers with mental health in such a context are necessary.

Further, currently, the evidence on the effect of mental health on health workers is predominantly quantitative. Although this form of evidence is important, it poses some limitations especially since it misses the social-cultural and lived experiences of the mental health of health workers. A qualitative investigation in this regard would unfold the contextual lived realities of healthcare workers regarding their mental wellbeing during this pandemic.

Therefore, taking the above two gaps into consideration, the aim of this study is to qualitatively investigate the lived experiences of mental health among frontline health workers providing COVID-19-realted care in Uganda. This study will provide insights into the contextual realities of the mental health of health workers facing greater challenges given the weak healthcare system. This is critical because for the COVID-19 response to be effective in such contexts, the mental health of healthcare providers must be factored in. The success of the response to COVID-19 ultimately depends on the efficiency of health care providers. Further, our study is informed by mounting array of studies that are focusing on mental health experiences and coping strategies among healthcare workers during the pandemic [2, 3]

## Main text

### Methods

#### Ethical consideration

Before commencement of the study, written ethical clearance was obtained from the University of Amsterdam (UvA) law school IRB and from the Ugandan local ethical clearance IRB called Makerere University School of Medicine Research and Ethics Committee (SOMREC). Besides that, our participants signed written consent in which they agreed to participate in our study. This was done after they were thoroughly informed of the objectives of the study. To ensure confidentiality, we anonymized the data and securely stored in our computer.

#### Study design

We conducted a cross sectional qualitative survey in the beginning of 2021. Our study focused on capturing healthcare workers who were based in the four major regions of Uganda (Northern, Eastern, Western and Southern). In order to do this, we relied on the AfriSight platform. This is a computerized that captures and maps out healthcare workers based on region in African countries. As we could not physically do the study in person given the COVID-19 restrictions, this platform set the basis of study.

#### Sample selection

The health worker participants were selected via the AfriSight database. We selected our sample randomly from the four regions of Uganda. Our participants included 50 healthcare workers. These were all involved in providing COVID-19-related care. See Table 1 for summary of demographics.

**Table 1:** Summary demographics

Variable	Observations	Variable levels	Frequency	Percentage of total (%)
Gender	50	Female	22	44
		Male	28	56
Job category	50	Clinical officer	2	4
		Doctor	29	58
		Laboratory personnel	1	2
		Nurse	14	28
		Others	2	4
		Pharmacist	2	4
Facility	50	District hospital	9	18
		Health centre III	2	4
		Health centre IV	5	10
		National referral	15	30
		Pharmacy	2	4
		Private clinic	8	16
		Regional referral	9	18
Facility ownership	50	Private owned	21	42
		Government/public owned	29	58

#### Data collection

Given the COVID-19-related restrictions, we collected our data using online survey tools hosted on the AfriSIght platform; for this purpose, we use the Qualtrics Survey Tool. The survey tool was created specifically for this study. The data collected related to the experience of mental health among healthcare workers, what the sources of health challenges was, what resources were available to handle these challenges, how did they cope. A sample questionnaire has been included as an appendix.

#### Analysis

After collecting the data, we imputed into NVivo software and relied on NVivo to analyze the data using thematic analysis strategy. Thematic analysis is a type of analysis that works by unearth themes imbedded in the data. This is done by building and organizing themes arising from the raw data in a systematic way. Thus, thematic analysis helped us systematically organize, examine and summarize our data based on the various emerging mental health themes. See Table 2.

**Table 2:** summary of qualitative results

Global theme	Codes
Types and sources of mental health disorders	Types: Depression Anxiety Posttraumatic disorders Causes: Long working hours Lack of proper equipment Lack of sleep Exhaustion
Coping strategies	Family networks Community networks Help from family Responsibility to society Assistance from community members Availability of assistance from strangers Symbiotic nature of assistance in community Increased rates of domestic violence

### Results and discussion

#### Types and sources of mental health problems reported

Our respondents generally reported three most common forms of mental health disorders which include depression, anxiety and post-traumatic disorder. Several respondents reported having experienced one of the three forms of mental health disorders. These findings are in line with what studies elsewhere have shown regarding what types and severity of mental health challenges among health workers during their fight against COVID-19 [2, 3, 6, 7]. Several Ugandan frontline healthcare workers highlighted that the sources of these disorders included long working hours, lack of proper equipment, lack of sleep, exhaustion, and experiencing high death rate under their care. The following sentiments from some of the health workers illustrates this.

*“Feelings of loss. Wishing I could have done more to save a patient, wishing resources were readily available to enable me do my work and save a life efficiently”*

*“We are working around the clock with little sleep and yet the cases keep increasing. Many of us are just exhausted and the public doesn’t quite get how overstretched we have been during this pandemic”*

*“Too many deaths and it looks like we are helpless. Bodies keep piling up, how can you sleep at night like that”*

Several studies are pointing out that depression, anxiety and post-traumatic disorder among healthcare workers have been exacerbated during this pandemic [8, 9]. The situation is not any different in Uganda. This has been linked to the fact that there has been less government support in expanding healthcare response in general and reflects what many scholars have long warned about the risks of poor healthcare systems in times of pandemics. The burden on healthcare workers is a reminder of the neglect health facilities have suffered over the years [5, 10]. Other studies also show that the poor state of the mental health of healthcare workers in Uganda has greatly affected the response to the pandemic [11, 12]. Failure to address the causes of mental health problems among frontline workers as observed above counters any sacrifices that many healthcare workers have been making since the start of the pandemic. This also further threatens the whole response strategy.

#### Coping strategies

Despite the high prevalence of mental health disorders among healthcare workers in Uganda, many demonstrate through their responses a type of agency and coping strategy that is fit for purpose and is imbedded in the cultural fabric of the country. Several of our participants highlighted how community and family networks were a useful source of support.

*“Talking to colleagues was useful for coping”*

*“Without my very supportive big family, it would have been difficult to continue in this job. Their prayers, help in terms of taking care of my chores and preparing food for me has been a huge boost for me..”*

*“In Uganda we are lucky to have a society that stands for each other. Neighbors, friends and strangers have really supported us in many ways during these struggles. Their help does not just end at clapping for us, they really physically show up when you need them..”*

Further, a sense a reciprocal sense of responsibility to society was also a motivating element for the continued effort. Our healthcare workers pointed out that they and the people they serve were ‘one’ meaning that their very survival is tied to the many efforts done by other society members. A symbiotic relationship in Ugandan society enables this.

*“Here we are one people, one family. We help each other in many ways. The farmers continue to play their part, the cleaners, ‘boda-boda’ drivers etc. They are all pushing their limits to make our society triumph under this scourge. We have a responsibility to them as well”*

Rather than boggling down under the veracity of mental health challenges, local health workers in Uganda relied on communal networks for support in order to get through their mentally challenging moments. A society with strong communal sensibilities and practices seemed to have provided avenues for healthcare workers to cope and be motivated to play their part. These findings present an opportunity of strengthening local responses by relying on feasible networks of survival. Further, as other studies have pointed out elsewhere within most resource poor settings, there exists “unrecognized and sometimes ignored portfolio assets” which are in most cases contextually profitable [13, 14]. These portfolio assets such as family and communal support have been used for generations and could serve as a useful coping strategy if scaled up (Additional file 1).

### Conclusion

All in all, our findings suggest that healthcare workers are under enormous stress during this pandemic, however, in order to effectively respond to the COVID-19 pandemic in Uganda, it is important to understand their challenges and sources of these challenges. The government thus has the reasonability to address most of the sources that were highlighted (long working hours, lack of proper equipment, lack of sleep, exhaustion, and experiencing high death rate under their care). Further, the Ugandan social fabric presents an opportunity for coping through its strong communal links and networks. Scaling these forms of local responses is cheap but contextually useful for a country with limited resources like Uganda.

## Limitations

Our study was conducted under Lockdown restrictions thus it was an online survey. This means our findings are only based only on the views of respondents who had access to WIFI and online platforms. This means that our results may have missed experiences of other people. However, despite all that, we posit that the study provides insightful findings and is a first step in understanding the views and lived experiences of healthcare regarding their mental health challenges during the COVID-19 pandemic in Uganda.

## Supplementary Information

**Additional file 1.** Interview Guide—Uganda.

## Data Availability

The datasets used and/or analysed during the current study available from the corresponding author on reasonable request.

## Abbreviations

COVID-19: Coronavirus Disease of 2019
GDPR: General Data Protection Regulation
HIV: Human immunodeficiency virus
SOMREC: Makerere University School of Medicine Research and Ethics Committee
SBIR: Small Business Innovation Research
WHO: World Health Organization
