# Supplementary material for: Exploring health workers’ experiences of mental health challenges during care of patients with COVID-19 in Uganda: a qualitative study
Source: BMC Res Notes. 2021 Jul 26;14:286. doi: 10.1186/s13104-021-05707-4 (PMC8312199; doi:10.1186/s13104-021-05707-4)
Supplement: Supplementary file 1 — Additional file 1. Interview Guide—Uganda. [file 13104_2021_5707_MOESM1_ESM.docx]

**Interview Guide***Mental health of frontline healthcare workers in Uganda*

**Introduction**
Welcome remarks and thank participants.

Introduce the study and allow them to sign the consent form

| Age |  |
| --- | --- |
| Gender |  |
| Job |  |
| Facility |  |
| Facility ownership | Facility owned by public/government or private |

**Part 1. Open ended Questions**

1) Have you suffered any mental health challenges (e.g stress, depression, anxiety, posttraumatic disorder etc.) during the COVID-19 pandemic, if yes, what exactly was it?

2) If you suffered a mental health challenge, what caused it?

3) If you suffered a mental health challenge, how did you cope with it?

4) Do you know any of your workmates who has suffered a mental health challenge (e.g stress, depression, anxiety, posttraumatic disorder etc.) during the COVID-19 pandemic, if yes, what exactly was it ?

5) If you know a workmate who suffered a mental health challenge, what caused it?

6) If you know a workmate suffered a mental health challenge, how did they cope with it?

**Part 2: Other Psychosocial Factors Associated With the pandemic**

1. Answer TRUE or False to the following questions:

| **Question** | **True** | **False** |
| --- | --- | --- |
| Generally, do you feel more stressed about the State of Emergency and State of Public Calamity measures? |  |  |
| Do you get upset by thinking about COVID-19? |  |  |
| Do you panic and overreact to news relating to COVID-19? |  |  |
| Are you worried about losses/your job when the State of Emergency or State of Public calamity measures are imposed? |  |  |
| Do you get worried about your family’s health more than usual? |  |  |

1. Are you sleeping more/less than you normally do?

- I sleep as usual
- I sleep more than usual
- I sleep less than usual

End of Interview
